# Correlation and comparative analysis of discriminative validity of the Scale of Oral Health Outcomes for Five-Year-Old Children (SOHO-5) and the Early Childhood Oral Health Impact Scale (ECOHIS) for dental caries

**DOI:** 10.1186/s12903-015-0021-y

**Published:** 2015-03-10

**Authors:** Izabella Barbosa Fernandes, Joana Ramos-Jorge, Maria Letícia Ramos-Jorge, Marcelo Bönecker, Jenny Abanto, Leandro Silva Marques, Saul Martins Paiva

**Affiliations:** Department of Pediatric Dentistry and Orthodontics, School of Dentistry, Federal University of Vales do Jequitinhonha e Mucuri, Rua Arraial dos Forros, 215, 39100-000 Diamantina, MG Brazil; Department of Pediatric Dentistry and Orthodontics, School of Dentistry, University of São Paulo, São Paulo, Brazil; Department of Pediatric Dentistry and Orthodontics, School of Dentistry, Federal University of Minas Gerais, Belo Horizonte, Brazil

**Keywords:** Quality of life, Oral health, Preschool children

## Abstract

**Background:**

The perceptions of parents and children regarding oral health are useful to oral public health and clinical practice in pediatric dentistry. The primary aim of the present study was to evaluate the correlation between the total and item scores of the Scale of Oral Health Outcomes for Five-Year-Old Children (SOHO-5) (parental version and child’s self-reports) and the Early Childhood Oral Health Impact Scale (ECOHIS). Subsequently, the discriminative validity of these assessment tools regarding dental caries was compared.

**Methods:**

One hundred twenty-one children randomly selected in the city of Diamantina (Brazil) were submitted to oral examinations. Parents answered the ECOHIS and SOHO-5p (parental version) and children answered the SOHO-5c (child’s self-reports). Statistical analysis involved the Mann–Whitney test as well as the calculation of Spearman’s correlation coefficients.

**Results:**

A significant correlation was found between the SOHO-5p and ECOHIS (r = 0.85), whereas no significant correlations were found between the SOHO-5c and SOHO-5p (r = 0.00) or between the SOHO-5c and ECOHIS (r = −0.41). Significant differences in the impact on quality of life were found between children with severe decay and no severe decay (caries free, with initial or established caries) both the ECOHIS and SOHO-5p (p ≤0.05), whereas no difference was found in SOHO-5c (p > 0.05).

**Conclusions:**

The ECOHIS and SOHO-5p were correlated with each other. The accounts of the children differed from their parents’ reports and were not capable of discriminating dental caries in advanced stages of progression.

## Background

The assessment of oral health-related quality of life (OHRQoL) is more difficult with preschool children due to their limited understanding of what is being evaluated [1]. As parents are responsible for their children’s wellbeing, it is important to explore their perceptions regarding the oral health of the children. Therefore, parents provide the best information in such cases [2].

In 2007, researchers at the University of North Carolina at Chapel Hill developed the Early Childhood Oral Health Impact Scale (ECOHIS) to be administered to parents and caregivers of preschool children. The ECOHIS has been tested and validated in the United States [3], Canada [4], China [5], Iran [6] and Brazil [7,8]. This scale furnishes valid, reliable information on the OHRQoL of preschool children through parental reports.

Based on evidence that children aged four to six years can reliably report on their own quality of life [9-11], the Scale of Oral Health Outcomes for Five-Year-Old Children (SOHO-5) was developed in the United Kingdom [12]. This scale has been translated into Portuguese, cross-culturally adapted and validated for use on Brazilian children aged five to six years [13]. The Brazilian version of the SOHO-5 has proven to be responsive to change and the authors suggest its use as an outcome indicator in clinical trials. Both the parental and the child versions have demonstrated satisfactory results [14]. However, due to the scarcity of studies, there is little evidence on the reliability and validity of the SOHO-5 or correlations between the reports of parents/caregivers and children.

Before the widespread use of an OHRQoL assessment tool, it is important to investigate its limitations, advantages and applications in different populations. Thus, the comparison between a recently developed assessment tool and one of proven validity is an adequate strategy for determining the quality of the former. It is also important to investigate the capacity of the assessment tool with regard to discriminating affected and non-affected individuals.

The primary aim of the present study was to evaluate the correlation between the total and item scores of the Scale of Oral Health Outcomes for Five-Year-Old Children (SOHO-5) (parental version and child’s self-reports) and the Early Childhood Oral Health Impact Scale (ECOHIS). Subsequently, the discriminative validity of these assessment tools regarding dental caries was compared among preschool children of five years of age.

## Methods

### Study population

A cross-sectional study was conducted with preschool children of five years of age and their parents/caregivers in the city of Diamantina, which is located in the northern portion of the state of Minas Gerais in southeastern Brazil. The inclusion criteria were age five years, enrolment in a preschool/daycare center in the city and parent/caregiver fluent in Brazilian Portuguese who lived with the child at least 12 hours per day. The exclusion criterion was any systemic disorder that could alter cognitive development, such as Down syndrome or cerebral palsy.

Five preschools (three public and two private) in the city of Diamantina were randomly selected for participation in the study. These schools had a total of 136 five-year-old students. The sample size was calculated using the formula for the estimate of linear correlation between two quantitative variables [15]. Considering a bilateral α of 0.05 and β of 0.10, 113 children would be needed to ensure that a correlation coefficient of 0.30 was significantly different from the null hypothesis. The hypothesis of this study was the existence of correlation between the instruments. To compensate for possible losses, all children aged five years at the preschools were recruited for the study. All parents were contacted through a letter explaining the objective of the study, along with a statement of informed consent authorizing their child’s participation. However, seven parents did not authorize their children’s participation. Thus, 129 pairs of children and parents/caregivers were included.

### Evaluation of impact on OHRQoL and socio-demographic data

Parents/caregivers were asked to answer the Brazilian versions of the SOHO-5[13] and ECOHIS [8] and fill out a questionnaire addressing socio-demographic data, such as mother’s schooling (years of study), household income (categorized based on the Brazilian monthly minimum salary = approximately US$ 310.00), type of school (public or private) and access to dental care. The children answered the child version of the SOHO-5 [13].

Both the child (SOHO-5c) and parent (SOHO-5p) versions of the SOHO-5 have seven items. The child version addresses difficulty eating, drinking, speaking, playing, sleeping and smiling due to dental problems. Three response options are provided (no = 0, a little = 1, a lot = 2) with the aid of a face scale. A trained examiner interviewed the children without the presence of the parents/caregivers to prevent their influence on the answers. The SOHO-5p was self-administered. Five response options are provided (not at all = 0, a little = 1, moderate = 2, a lot = 3, a great deal = 4); a “don’t know” option is included, which is not scored. The total score ranges from 0 to 14 in the child version (SOHO-5c) and from 0 to 28 in the parents version (SOHO-p).

The ECOHIS has 13 items distributed between the Child Impact and Family Impact sections. The scores are calculated based on a five-point Likert scale with response options that range from “never” (0 points) to “very often” (4 points). The total score ranges from 0 to 52 [Child Impact section – symptoms: 1 item (range: 0 to 4); function: four items (range: 0 to 16); psychology: 2 items (range: 0 to 8); self-image/social interaction: two items (range: 0 to 8); Family Impact section – parental distress: two items (range: 0 to 8); family function: two items (range: 0 to 8)].

The total score of both the SOHO-5 and ECOHIS is calculated by the sum of the codes for each item, with higher scores denoting a greater negative impact on quality of life.

The children were interviewed first. Subsequently, the SOHO-5p and ECOHIS questionnaires were sent to the parents/caregivers one week apart to be filled out at home. The aim of the one-week interval between questionnaires was to avoid the influence of one on the responses of the other. The ECOHIS was sent first.

### Oral examination

To evaluate the discriminative validity of the questionnaires, the presence and stage of untreated dental caries were investigated. This condition was chosen due to its association with the quality of life of preschool children. The clinical exam was performed by an examiner who had undergone a training and calibration exercise using the criteria of the International Caries Detection and Assessment System (ICDAS II) [16]. The calibration exercise was performed at a public preschool with a sample of 80 children for the calculation of inter-examiner kappa coefficients. Fifty children were examined a second time after a one-week interval for the calculation of the intra-examiner kappa coefficients. The calculation of kappa was performed considering the worst condition of each tooth. All kappa coefficients were greater than 0.80. The oral exam was performed after brushing by the examiner with the aid of a headlamp (PETZL®, Tikka XP, Crolles, France), mouth mirror (PRISMA, São Paulo, SP, Brazil), World Health Organization probe (Golgran Ind. e Com. Ltda., Sao Paulo, SP, Brazil) and dental gauze to dry the teeth. All equipment was previously sterilized. During the exam, the child laid on a portable cot.

The ICDAS II was used to determine the stage of dental caries. The first visual change in enamel (code 1) is frequently detected only after drying with compressed air. As drying was performed with dental gauze in this study, the decision was made to exclude code 1 from the evaluation. The distinct visual change in enamel (code 2) was considered ‘early stage decay’. The localized enamel breakdown (code 3) and underlying dark shadow from dentin (code 4) were considered ‘established decay’. ‘Severe decay’ was recorded in the case of dental caries with a distinct cavity and visible dentin (code 5) or extensive distinct cavity with visible dentin (code 6).

### Data analysis

Data analysis was performed with the aid of the Statistical Package for Social Sciences (SPSS for Windows, version 20.0, SPSS Inc. Chicago, IL, USA) and involved descriptive analysis for the socio-demographic data, caries stage and total ECOHIS and SOHO-5 scores. Dental caries was classified by the worst condition found in child. If a tooth had both a white spot and dentinal lesion, the tooth was classified by the latter condition. Children with severe decay were compared with children without severe decay in relation to OHRQoL evaluated by SOHO-5p, SOHO-5c and ECOHIS. The Kolmogorov-Smirnov test was used to determine the distribution of the quantitative variables (normal or non-normal). Since this distribution was non-normal (all p < 0.001), the Mann Whitney test was used to analyze the discriminative validity (difference between children with severe decay and children without severe decay) of each questionnaire in relation to the dental caries, using a significance level of 5%. Furthermore, the effect size was investigated. The calculation of the effect size proposed by Cohen was used to test the clinical significance of the results. Based on Cohen’s criteria, an effect size < 0.2 indicates a difference of small magnitude, 0.2 to 0.7 indicate a moderate difference and > 0.7 indicates a large difference. Spearman’s correlation coefficients were calculated to determine the strength of the following correlations: SOHO-5p vs. SOHO-5c; SOHO-5p vs. ECOHIS; and SOHO-5c vs. ECOHIS. All items from SOHO-5p and SOHO-5c were considered for this analysis. Since ECOHIS has nine items on the child impact section, seven items similar to the SOHO-5 were chosen. For total ECOHIS was performed the sum of the scores of the seven items used. The internal consistency (Cronbach’s alpha) of ECOHIS, SOHO-5p and SOHO-5c were greater than 0.90.

### Ethical considerations

This study received approval from the Human Research Ethics Committee of the Federal University of Minas Gerais, Belo Horizonte, Brazil (protocol number 09066012.3.0000.5149). All parents/caregivers signed a statement of informed consent.

## Results

### Characteristics of participants

One hundred twenty-nine five-year-old children and their parents/caregivers participated in the present study and 121 parents/caregivers (93.8%) returned the completed questionnaires. No questionnaire was excluded from the analysis due to incomplete data. No parent/caregiver answered “don’t know” to any of the items. Most questionnaires were filled out by the mothers (85.9%).

A total of 49.6% of the children were caries free, 8.3% had early stage decay, 14% had untreated established decay and 28.1% had untreated severe decay. Moreover, the children had different socio-demographic characteristics (Table 1).

**Table 1:** **Socio-demographic aspects of children and families (n = 121)**

	**Without severe decay**	**With severe decay**	p*	**d**
	**n (%)**	**n (%)**		
Sex of child				
Female	44 (66.7)	22 (33.3)	0.161	0.128
Male	43 (78.2)	12 (21.8)		
Mother’s schooling				
>8 years	15 (93,8)	1 (6.2)	0.033	0.207
4 to 8 years	52 (71.2)	21 (28.8)		
<4 years	20 (62.5)	12 (37.5)		
Household income				
<1 to 2 times monthly minimum salary	35 (92.1)	3 (7.9)	<0.001	0.347
2 to 3 times monthly minimum salary	25 (73.5)	9 (25.5)		
4 to 15 times monthly minimum salary	27 (55.1)	22 (44.9)		
Type of school				
Public	18 (100.0)	0 (0.0)	0.004	0.261
Private	69 (67.0)	34 (33.0)		
Access to dentist				
Yes	52 (89.7)	6 (10.3)	<0.001	0.379
No	35 (55.6)	28 (44.4)		

*Chi-square test; d: Effect size Cohen's.

### OHRQoL instruments

The SOHO-5p score ranged from 0 to 16 (mean: 1.9 ± 3.9); the SOHO-5c score ranged from 0 to 14 (mean: 1.9 ± 3.3); and the ECOHIS score ranged from 0 to 31 (mean: 3.9 ± 6.8). The frequency of impact on quality of life (SOHO-5 > 0) was 64% according to parents/caregivers and 37.2% according to the children. Using the ECOHIS, 40.5% of parents/caregivers reported impact on the quality of life of the children (ECOHIS > 0). Moreover, parents reported a greater frequency of impact related to the child (38.0%) than the family (24.0%).

### Correlations: SOHO and ECOHIS

No significant correlations were found among the answers of the children on the SOHO-5c and the answers of the parents/caregivers on the SOHO-5p or ECOHIS (all p > 0.05) (Table 2). However, significant correlations were found among the answers of the parents on the SOHO-5p and ECOHIS (p < 0.001).

**Table 2:** **Responses to SOHO-5p, SOHO-5c and ECOHIS questionnaires**

	**SOHO-5 parents vs. ECOHIS**	**SOHO-5 child vs. SOHO-5 Parents**	**SOHO-5 child vs. ECOHIS**
	**r**	**R**	**r**
Difficulty eating	0.89*	0.19	−0.00
Difficulty drinking	-	-	−0.00
Difficulty speaking	0.91*	−0.29	−0.09
Difficulty playing	-	−0.09	-
Difficulty sleeping	0.57*	−0.08	−0.09
Avoided smiling (due to pain)	0.66*	−0.13	−0.10
Avoided smiling (due to appearance)	0.55*	0.00	−0.11
**Total score**	0.85*	0.00	−0.41

r: Spearman’s correlation coefficient; *Significant correlation (p ≤ 0.05).

### OHRQoL instruments and dental caries

No significant difference was found between children with severe decay and without severe decay in the SOHO-5c score (all p ≥ 0.05) (Table 3). However, significant differences were found between two groups both the SOHO-5p score and ECOHIS score (p < 0.05) (Table 3). With the exception of the ECOHIS Family Function subscale, significant difference were found between the stage of dental caries and each item of the questionnaires, with higher scores among children with more severe caries. In general, the effect size for the SOHO-5c were lower than the SOHO-5p and ECOHIS.

**Table 3:** **Discriminative validity of SOHO-5c, SOHO-5p and ECOHIS according to presence or absence of severe decay**

	**Without severe decay**		**With severe decay**		**p-value** ^*^	**d**
	**Mean (SD)**	**CI 95%**	**Mean (SD)**	**CI 95%**		
**SOHO-5 child**						
Difficulty eating	0.5 (0.7)	(0.33-0.64)	0.5 (0.8)	(0.25-0.80)	0.837	0.06
Difficulty drinking	0.4 (0.6)	(0.24-0.51)	0.4 (0.7)	(0.15-0.67)	0.902	0.04
Difficulty speaking	0.2 (0.5)	(0.11-0.33)	0.3 (0.7)	(0.08-0.56)	0.574	0.17
Difficulty playing	0.9 (0.3)	(0.01-0.15)	0.1 (0.5)	(0.03-0.32)	0.525	0.16
Difficulty sleeping	0.3 (0.6)	(0.15-0.42)	0.4 (0.8)	(0.14-0.68)	0.524	0.17
Avoided smiling (due to pain)	0.3 (0.6)	(0.14-0.39)	0.1 (0.5)	(0.03-0.32)	0.931	0.21
Avoided smiling (due to appearance)	0.2 (0.5)	(0.10-0.32)	0.2 (0.6)	(0.02-0.45)	0.179	0.05
**Total score**	1.9 (3.2)	(1.23-2.58)	2.2 (3.7)	(0.90-3.51)	0.690	0.09
**SOHO-5 parents**						
Difficulty eating	0.2 (0.6)	(0.07-0.32)	0.9 (1.0)	(0.60-1.28)	<0.001	0.90
Difficulty speaking	0.0 (0.1)	(0.01-0.06)	0.5 (0.8)	(0.20-0.80)	<0.001	0.69
Difficulty playing	0.0 (0.2)	(0.01-0.10)	0.4 (0.7)	(0.17-0.66)	<0.001	0.68
Difficulty sleeping	0.0 (0.2)	(0.01-0.10)	0.6 (0.9)	(0.34-0.96)	<0.001	0.92
Avoided smiling (due to pain)	0.2 (0.5)	(0.05-0.27)	1.2 (1.0)	(0.91-1.62)	<0.001	1.35
Avoided smiling (due to appearance)	0.1 (0.3)	(0.01-0.13)	0.5 (0.8)	(0.25-0.80)	<0.001	0.77
Self-confidence/self-esteem	0.1 (0.3)	(0.01-0.17)	0.8 (1.0)	(0.51-1.20)	<0.001	1.03
**Total score**	0.7 (2.3)	(0.24-1.23)	5.1 (5.4)	(3.22-7.02)	<0.001	1.05
**ECOHIS**						
**Child impact section**						
***Symptoms***	0.2 (0.6)	(0.09-0.34)	2.0 (1.2)	(1.59-2.41)	<0.001	1.91
Oral/dental pain						
***Function***	0.1 (0.4)	(0.00-0.19)	0.7 (0.9)	(0.40-1.07)	<0.001	0.87
Difficulty drinking						
Difficulty eating	0.2 (0.6)	(0.05-0.29)	0.9 (1.1)	(0.53-1.29)	<0.001	0.86
Difficulty pronouncing words	0.1 (0.5)	(0.01-0.20)	0.2 (0.5)	(0.06-0.41)	0.003	0.30
Missed preschool or school	0.0 (0.2)	(0.00-0.07)	0.3 (0.6)	(0.14-0.56)	<0.001	0.73
***Psychological***	0.0 (0.2)	(0.01-0.10)	0.7 (1.0)	(0.36-1.06)	<0.001	1.24
Trouble sleeping						
Irritable or frustrated	0.0 (0.2)	(0.01-0.10)	0.9 (1.1)	(0.50-1.26)	<0.001	1.05
***Child self-image/social interaction***	0.1 (0.5)	(0.02-0.21)	0.9 (1.2)	(0.50-1.38)	<0.001	0.88
Avoided smiling or laughing						
Avoided talking	0.0 (0.1)	(0.01-0.03)	0.7 (1.1)	(0.30-1.06)	<0.001	0.87
**Family impact section**						
***Parental distress***	0.1 (0.4)	(0.03-0.22)	1.2 (1.5)	(0.69-1.72)	<0.001	0.99
Been upset						
Felt guilty	0.2 (0.6)	(0.04-0.32)	1.3 (1.5)	(0.79-1.86)	<0.001	0.97
***Family function***	0.1 (0.5)	(0.02-0.18)	0.2 (0.7)	(0.07-0.43)	0.229	0.16
Time off from work						
Financial impact	0.1 (0.4)	(0.03-0.20)	0.2 (0.8)	(0.02-0.55)	0.480	0.23
**Total score**	1.4 (3.2)	(0.66-2.05)	10.3 (8.9)	(7.19-13.40)	<0.001	1.33

*Mann–Whitney test; d: Effect size Cohen's.

## Discussion

Unlike the ECOHIS and SOHO-5p, the SOHO-5c was unable to discriminate between the presence of severe decay and absence of severe decay among five-year-old children. In the present study, the decision was made only to evaluate discriminative validity because the scales employed have already been validated for Brazilian Portuguese [7,8,12,17]. Moreover, despite the SOHO-5c have shown high value of Cronbach’s alpha (0.90), as well as SOHO-5p and ECOHIS (Cronbach’s alpha 0.93 and 0.94, respectively), no correlations were found among the answers of the children on the SOHO-5c and the answers of the parents/caregivers on the SOHO-5p or ECOHIS.

The inability of the SOHO-5c to discriminate children with and without severe decay in the present study underscores the challenge of evaluating the perceptions of preschool children with regard to OHRQoL. These findings are also supported by the low effect size found in SOHO-5c. Previous studies have demonstrated that a child’s perception regarding his/her oral health is influenced by age, cognitive development, emotional development and the social context in which the child lives [1,18]. The age of six years marks the onset of abstract thinking, the construction of one’s self-image, the capacity to understand basic health concepts and the ability to recall past events [19-21]. Thus, it is likely that the five-year-olds surveyed in the present study did not have sufficient cognitive or emotional development to understand, interpret and answer the SOHO-5 in a valid fashion.

On the other hand, a number of studies report that preschool children are able to report on their OHRQoL in a valid, reliable manner using the SOHO-5c [12,13]. A Brazilian study carried out to test the responsiveness of the SOHO-5 found a better performance on the SOHO-5c [14]. However, it is important to consider the sample on which the scale was tested in these studies. The study on the development of the scale was conducted in the United Kingdom [12] and social context can exert an influence on the cognitive development of children [18]. While the Brazilian studies [13,14] involved samples with a similar social context as that of the present investigation, both five-year-olds and six-year-olds were evaluated in a clinical setting, whereas the scale was developed only for five-year-olds. Thus, caution must be used when interpreting the results, as six-year-olds have a greater capacity for understanding and answering questions due to their more advanced cognitive development [20]. Moreover, the fact that the majority of children in the sample studied at a public school may be an indicator of a low socioeconomic status, which could probably limit children’s understanding of the questionnaire. A number of studies report that socioeconomic factors exert a direct influence on answers regarding the impact of different adverse health conditions on quality of life [22,23]. Therefore, further studies should be conducted with children from different social classes.

The association between caries experience and the reports of parents/caregivers through the ECOHIS and SOHO-5p substantiates evidence that parents/caregivers can provide valid, reliable information regarding the OHRQoL of their preschool children [3,20,24,25]. Unlike the present investigation, a previous study conducted in Brazil found significant correlations in the reports of parents/caregivers and their children using the SOHO-5. However, the study included six-year-olds in the sample and did not evaluate the discriminative power of the scale with regard to oral health problems.

The prevalence of impact from dental caries on the OHRQoL of the children, as determined using the SOHO-5p, was similar to the rate reported in the validation study for the Brazilian version of this scale [13]. However, the prevalence rate was greater than that found when using the ECOHIS in the present investigation. Further studies should be conducted to determine whether the difference in the prevalence of impact between the two questionnaires (SOHO-p and ECOHIS) is due to the way the items are written and the expressions used in the answers, which may influence the understanding on the part of respondents.

The total SOHO-5p and ECOHIS scores demonstrate the ability to distinguish between children with severe caries and those without severe decay. These findings are in agreement with data reported in previous studies, which found an association between severe caries (as detected using the ICDAS II) and an impact on the OHRQoL of preschool children [26,27].

The effect size was congruent with the p-value in the present study. The effect size has greater clinical significance when there is a mild, moderate, high or no effect, whereas the statistical test of the null hypothesis only determines whether a given association is significant or not. It is possible for the statistical test of the null hypothesis to indicate a lack of differences between groups due to the heterogeneity of the participants, whereas the effect size may be high. Moreover, considering p < 0.05 as indicative of statistical significance, p = 0.056 would be rejected, whereas the effect size of these two values (0.05 and 0.056) could be the same. Hence, the effect size is a more precise, less arbitrary criterion [28].

Although the present study provides substantial original evidence, further studies involving the SOHO-5 are needed. Such studies should involve representative samples of five-year-old children from different populations and social contexts to establish the discriminative properties of this scale more reliably. Moreover, test-retest reliability was not evaluated.

## Conclusions

The ECOHIS and SOHO-5p demonstrated similar capacity for the evaluation of OHRQoL among preschool children. Both questionnaires proved capable of distinguishing between children with severe caries and those with no caries experience or caries in the less advanced stages. However, the reports of children differed from the reports of their parents/caregivers and the SOHO-5c was unable to discriminate children with and without caries.
